# Vehicle Behavior Discovery and Three-Dimensional Object Detection and Tracking Based on Spatio-Temporal Dependency Knowledge and Artificial Fish Swarm Algorithm

**DOI:** 10.3390/biomimetics9070412

**Published:** 2024-07-06

**Authors:** Yixin Chen, Qingnan Li

**Affiliations:** 1 School of Artificial Intelligence, Jianghan University, Wuhan 430056, China; 2 Engineering Research Center for Transportation Systems, Wuhan University of Technology, Wuhan 430070, China; qingqing@whu.edu.cn

**Keywords:** 3D object detection, 3D object tracking, convolutional neural networks, knowledge-based vehicle behaviors discovery, artificial fish swarm algorithm

## Abstract

In complex traffic environments, 3D target tracking and detection are often occluded by various stationary and moving objects. When the target is occluded, its apparent characteristics change, resulting in a decrease in the accuracy of tracking and detection. In order to solve this problem, we propose to learn the vehicle behavior from the driving data, predict and calibrate the vehicle trajectory, and finally use the artificial fish swarm algorithm to optimize the tracking results. The experiments show that compared with the CenterTrack method, the proposed method improves the key indicators of MOTA (Multi-Object Tracking Accuracy) in 3D object detection and tracking on the nuScenes dataset, and the frame rate is 26 fps.

## 1. Introduction

Three-dimensional object motion detection and tracking is an important basic component of autonomous driving technology. Famous autonomous driving datasets such as nuScenes [1], KITTI [2], and apolloscape [3] all contain 3D object motion detection and tracking tasks. Importantly, 3D object motion detection and tracking provides more environmental perception details than 2D moving object detection and tracking, which can better help autonomous vehicles perceive the surrounding environment and avoid collisions. 

Three-dimensional object motion detection and tracking has a certain robustness in a non-occlusion environment. However, with the increasing complexity of the road traffic environment, targets are often occluded by moving objects, such as vehicles and pedestrians, or stationary objects, such as road signs and trees. During the occlusion period, not only does the distribution of target appearance features change greatly, but the target motion features are also prone to complex personalized variation due to the interference of neighboring moving objects. How to accurately detect and stably track the 3D bounding box of the target in the occlusion period and, after getting rid of the occlusion, determine its size, position, and orientation and assist the autonomous driving system in adopting the appropriate driving strategy during the occlusion period to ensure driving safety is a key problem that urgently needs to be solved in the field of 3D target detection and tracking.

**Figure 1:**
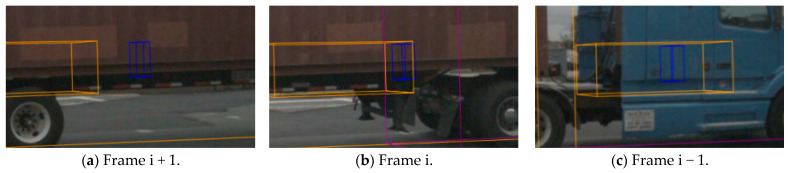
In severe-occlusion scenes, the target object is usually subjected to prolonged continuous severe occlusion, during which the discrimination of appearance and motion features is significantly decreased.

The CenterTrack [1] method designs a target object detection branch network and an offset prediction branch network and uses a distance-based greedy algorithm to associate the target objects between the previous and next frames. It achieves excellent performance on nuScenes and KITTI datasets. However, in a complex-occlusion scene caused by neighboring moving objects, the existing methods usually have three obvious shortcomings:

1. The apparent features of the target object change significantly between frames, and the large variation in the feature distribution law leads to a large search space for feature matching between frames [4].
2. In the complex motion scene of the target vehicle, the behaviors of the target vehicle and various interfering objects are intertwined. The target vehicle motion driving law changes complexity during occlusion, which leads to a sharp decline in tracking accuracy [5].
3. In severe-occlusion scenarios, the target vehicle is usually subjected to a long period of continuous severe occlusion, and the discrimination between its previous apparent features and motion features is reduced, resulting in a decrease in tracking accuracy [6], as shown in Figure 1.

In response to these problems, we first proposed three methods below:

1. We propose a graph neural network-based method with multi-feature learning to explore the coordinated change law of the target object’s position, direction, and speed; mine the spatio-temporal dependence knowledge in the coordinated change of the target object’s motion; and improve the graph node aggregation feature discrimination.
2. We propose a graph neural network-based method with multi-source feature interweaving, exploring the interweaving motion law of the target object and neighboring moving objects and iteratively updating the graph node features through multi-source feature aggregation to mine the relationship between neighboring data samples.
3. Finally, a graph neural network-based method with environmental interaction and collaboration is proposed to explore the interaction and collaboration rules between the target object and the traffic environment. The proposed method can accurately calibrate and stably track the target object under severe or continuous occlusion.

The main contributions of this study are as follows:

1. Starting from the simple motion of the target object, the potential geometric consistency in the rotation and translation transformation of the target is found, and the target structure knowledge and context information are shared. Compared with the appearance features in occluded scenes, the latent geometric consistency features based on rotation and translation transformation are more discriminative.
2. Using the complex motion of the target object, the interweaving motion behavior between the target and its neighboring interference objects is studied. Using the previous motion data of the target, the knowledge of the target motion behavior is mined, and the motion trajectory of the target during the occlusion period is constrained, which is helpful to stably track the 3D bounding box of the target during the occlusion period and, after getting rid of the occlusion, to accurately determine its size, position, and orientation. It can provide reliable motion planning clues for automatic driving systems and auxiliary driving systems to ensure driving safety.

## 2. Objectives

At present, the research work in the field of 3D object motion detection and tracking can be roughly divided into two categories according to the type of data collection: one type of data comes from lidar, which actively emits laser beams and receives signals to realize efficient detection of the surrounding environment. The generated point cloud data has a 360-degree field of view and precise ranging. Therefore, the method based on lidar data is more reliable in extreme lighting environments such as night and bright light but in severe weather conditions, such as haze, rain, snow, and so on, it is easily affected by dust and water droplets in the air [7]. One kind of data comes from the camera, and the light information is captured by the camera's imaging system. The generated image contains the shape and texture information of the object. Therefore, the method based on image data has certain robustness in scenes such as when the object is occluded [8,9], but the performance of the algorithm is affected under conditions such as low light, and the object has no texture [7]. With the development of deep learning, the research on image-based 3D object motion detection and tracking is mainly divided into two categories: one is 3D object detection based on single-view (monocular) images, and the other is 3D object detection and tracking based on multi-view images (including stereo image data and video data).

## 3. Related Work

### 3.1. Three-Dimensional Object Detection from Single-View Images

In the complex-occlusion scene caused by neighboring moving objects, the apparent features of the target object change significantly between frames, and the feature distribution law of the target object changes greatly. Therefore, some researchers use deep convolutional neural networks to extract the local features of objects, learn local–global spatial structure knowledge of objects, and reason about global information about objects to realize 3D object-detection tasks. Importantly, 3D object detection based on single-view images can be divided into two subcategories: 3D object-detection methods based on region-selection networks (RPNs) and 3D object-detection methods based on keypoint prediction.

The 3D object-detection method based on the region-selection network improves the performance of 3D object detection [10,11] by detecting the 2D selection box of the object [12], reasoning about the 3D edge box of the object and the 3D coordinates of the object parts [13], and predicting the pose and shape of the object in the 3D space [14]. However, this kind of method extracts and classifies the features of each potential object candidate box and calculates the Intersection over Union (IoU) ratio to remove the duplicate detection boxes of the same object. Such 3D object-detection methods using a post-processing step of non-maximum suppression are classified as two-stage detection methods, which are usually not end-to-end trainable and suffer from a lot of computational redundancy.

The 3D object-detection method based on key points converts the object-detection task into a standard key point prediction problem [8]. The convolutional neural network is used to extract the input image features and generate the key point heat map, where the peak point of the heat map is the center point; finally, the object attributes are predicted through the image features around the center point. Since there is no post-processing step, these methods are classified as one-stage detectors, which are more efficient than two-stage detectors and have a good trade-off between speed and accuracy.

### 3.2. Three-Dimensional Object Detection and Tracking from Multi-View Images

The research of 3D object detection and tracking based on multiple views can be divided into two categories: one is 3D object detection based on stereo image data, and the other is 3D moving object detection and tracking based on an image sequence.

Three-dimensional Object Detection Based on Stereoscopic image data

Using the 3D object detection framework, the network structure of the planesweep volume and 3D geometric volume is designed to determine the pixel correspondence constraints in the left and right views. Then, the corresponding constraint transformation of pixels in the planar scanning volume is connected to the 3D geometry to encode 3D information and semantic clues, and the accurate detection of 3D bounding boxes in a regular 3D space is realized [15]. Paired images taken by stereo cameras contain additional depth information compared to single-view images. The 3D object-detection methods based on stereo images usually encode the left view and right view, analyze and process the depth information, reason about the semantic and geometric information of the object, and realize the accurate detection and calibration of the 3D bounding box.

nuScenes [1], KITTI [2], and other autonomous driving datasets contain video sequence data for 3D object motion detection and tracking. Among them, an image sequence in the uScenes dataset contains 40 keyframes, and an image sequence in the KITTI dataset contains 4 keyframes. In the method of 3D object motion detection and tracking based on the image sequence, the single-view image and stereo image contain the appearance features of the object at the current time, and the image sequence contains the spatio-temporal information of each object in the road traffic scene. Analyzing and processing this information is helpful to improve the performance of 3D object motion detection and tracking.

The artificial fish swarm algorithm (AFSA) is an intelligent optimization method based on fish aggregation, foraging, following, and random behavior. The algorithm improves its own state according to the current food concentration perception and distribution of partner fish school position bulletin board status. Through this behavior selection, artificial fish will eventually continue to converge to obtain a better extreme value. The artificial fish swarm algorithm can adapt to two-dimensional and three-dimensional environments well, and it has low solving requirements and strong solving ability. It can run efficiently in complex environments and can be integrated with other algorithms to make up for the shortcomings so as to achieve more optimized solutions.

In summary, there are two main image-based 3D object-detection and tracking methods. One is to use a deep neural network to extract object features in each frame of the video for detection and matching. The other is to use a deep neural network to encode spatio-temporal information of image sequences. In the complex-occlusion scene caused by neighboring moving objects, the apparent features of the target object change significantly between frames. The first method usually has difficulty in extracting the apparent features with high discrimination, and the large variation in the feature distribution leads to a large search space for feature matching between frames, which affects the accuracy of motion detection and tracking. In the second method, in the complex-motion scene, the personalized motion of the target vehicle is interwoven with the behavior of its neighboring interference objects, and its motion-driving law has a complex personality in the occlusion period. A mutation results in a sharp decline in tracking accuracy. In severe-occlusion scenarios, the target vehicle is usually severely occluded for a long time, and the discrimination between its previous appearance features and motion features is reduced. As a result, there may be a variety of personalized motion laws for the target vehicle during the continuous-occlusion period, which restricts the tracking accuracy. Starting from the simple-motion scene of the target object, it is urgent to study the constraint mechanism of the potential geometric continuity and appearance characteristics of the object translation and rotation transformation between frames to narrow the search space and reduce the error. For complex-occlusion scenes, it is urgent to study the previous driving data of each object to find the complex motion habits of the target object and constrain its driving trajectory during the occlusion period according to them, so as to improve the target tracking accuracy. For severe-occlusion scenes, it is urgent to study the collaborative calibration method of vehicle forward and reverse motion trajectories. The algorithms in References [16,17] have problems in occluded scenes and do not perform well. To solve this problem, we study the characteristics of the scene, and based on the characteristics, we improve the Swarm Fishing Algorithm. Finally, it was used to further optimize the motion path.

### 3.3. 3D Object Detection and Tracking in Complex-Occlusion Scenes

The overall research of this dissertation is divided into three sequential levels. For simple-motion scenes, the latent geometric continuity of translation and rotation transformation of vehicle objects between frames is explored, and the latent geometric continuity appearance model is studied. For complex motion scenes, through the previous driving data of each vehicle, the complex motion habits of the target vehicle are mined, and the driving trajectory of the target vehicle within the occlusion period is constrained according to the complex motion habits. For the severely occluded scene, the motion trajectory of each vehicle in the occlusion period is inversely estimated, and the motion trajectory of the target vehicle is matched with the forward estimated trajectory of the target vehicle, and the artificial fish swarm algorithm is used to further optimize the motion path. The specific research content includes the following aspects:

1. Apparent models of latent geometric continuity;

Researchers have proposed various global feature or local feature appearance models based on single or multiple keyframe images to represent road users such as vehicles. However, in complex-occlusion scenes caused by nearby moving objects, the appearance features of target objects change significantly between frames. It is always difficult to effectively overcome the problem wherein the large variation in feature distribution leads to a large search space of feature matching between frames. In the field of key point detection, in order to ensure the accurate prediction of key points occluded by the object itself, researchers have explored the potential geometric continuity of key points between two frames, designed a two-stream deep convolutional network to extract the key point features of the same object under different viewpoints at the same time, and constructed the geometric constraints of key point spatial transformation to achieve the accurate prediction of invisible key points [18]. Inspired by this idea, this study introduces the continuity assumption of translation and rotation transformation between video frames, explores the constraint mechanism between latent geometric continuity and appearance features, and establishes the latent geometric continuity appearance model to reduce the error introduced by a large search space.

2. Complex motion habit Mining and driving trajectory constraint method;

The existing 3D object-motion-detection and -tracking methods based on video data usually use the appearance features of the target object and the past vehicle trajectory to detect and track the motion of the target object. However, in complex motion scenes, the motion of the target vehicle is intertwined with the behavior of the interference object, and its motion driving law changes complex during occlusion, resulting in a decrease in tracking accuracy. In recent years, faced with the rapid growth of video data, understanding and analyzing video data to solve problems such as action recognition, abnormal event detection, and activity understanding have become research hotspots in the field of computer vision video analysis. Researchers have used 3D convolution kernels to extract temporal and spatial features of video data, capture object motion information in video streams, and calculate high-level motion features to assist the output to enhance model performance and identify abnormal human behaviors [19]. Inspired by this idea, this study uses the past driving data of each vehicle to find the complex motion habits of the target vehicle and constrain its driving trajectory during the occlusion period so as to improve the target tracking accuracy.

3. Collaborative calibration of vehicle forward and reverse motion trajectories and artificial fish swarm algorithm.

In severe-occlusion scenarios, the target vehicle is usually severely occluded for a long time, and the discrimination between its previous appearance features and motion features is reduced. As a result, there may be a variety of personalized motion laws for the target vehicle during the continuous-occlusion period, which greatly restricts the tracking accuracy. In the field of video coding, video coding standards such as H.264 [20] and HEVC [21], such as bidirectional predictive interpolation coding frame, realize efficient compression of video temporal redundant information by using its preceding frame and following frame. Inspired by this idea, the motion trajectories of each vehicle during the occlusion period are inversely estimated after the target vehicle is out of occlusion, and the forward trajectory of the target vehicle is estimated by matching calculation so as to realize the collaborative calibration of the target vehicle’s motion trajectory. Finally, the artificial fish swarm algorithm is used to optimize the tracking results.

The research objectives are to realize the latent geometric continuity appearance model to meet the requirements of autonomous driving in complex-occlusion scenes, mining complex motion habits and driving trajectory constraints, and collaborative calibration of vehicle forward and reverse motion trajectories so as to improve the accuracy of 3D object motion detection and tracking. These include the following:

1. The continuity assumption of translation and rotation transformation behavior of vehicle objects between video frames is introduced, the constraint mechanism between latent geometric continuity and appearance characteristics is explored, and the latent geometric continuity appearance model is established to reduce the error introduced by the large search space;
2. Using the past driving data of each vehicle, the complex motion habits of the target vehicle are mined, and its driving trajectory during the occlusion period is constrained accordingly to improve the target tracking accuracy;
3. After the target vehicle gets rid of the occlusion, the motion trajectory of each vehicle in the occlusion period is estimated in the reverse direction and matched with the forward estimated trajectory of the target vehicle. The tracking result is optimized by combining the artificial fish swarm algorithm.

## 4. Materials and Methods

**Figure 2:**
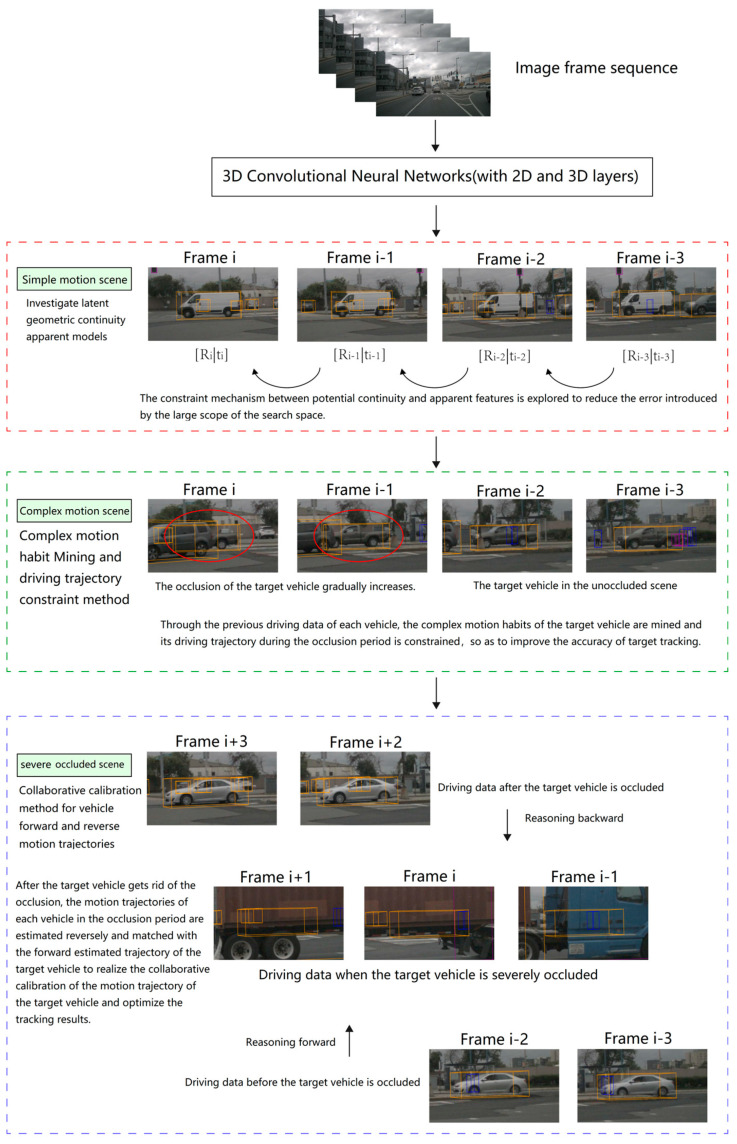
Framework of 3D object motion detection and tracking.

In this study, theoretical analysis and experimental verification are combined to carry out the research on 3D object motion detection and tracking in complex-occlusion scenes. Figure 2 shows the research framework. Work 1: Study latent geometric continuity appearance model. Work 2: Study complex motion habit mining and driving trajectory constraint method; Work III: Study the collaborative calibration method of vehicle forward and reverse motion trajectories. The specific research methods adopted in each work are shown in Figure 2. In the figure, the red circle represents the occluded target in the complex motion scene.

### 4.1. Investigate Latent Geometric Continuity Apparent Models

In the complex-occlusion scene caused by neighboring moving objects, the apparent features of the target object change significantly between frames, and the large variation of the feature distribution leads to the large search space of feature matching between frames. Traditional methods mainly focus on global or local feature representation based on single or multiple keyframe images. However, these single-point features discard a lot of continuous apparent information about the object sequence, which is easily disturbed by the motion of the vehicle and its nearby objects and affects the performance of 3D object motion detection and tracking.

In the field of keypoint estimation, the potential geometric continuity of keypoints under different viewpoints provides us with a useful idea. Specifically, in order to overcome the loss of local features caused by the occlusion of invisible keypoints by the object itself, researchers propose a two-stream keypoint prediction framework. Unlike traditional methods that directly regression keypoints through heat maps, this method simultaneously extracts local features of the same object keypoints under different viewpoints and then constructs a keypoint spatial geometric constraint model [18]. A good keypoint prediction effect is achieved. The reason is that the same keypoint has potential geometric continuity under different viewpoints. Therefore, the relative loss function of latent geometric continuity based on the translation and rotation transformation of key points between frames can more accurately describe the spatial transformation of object key points under different viewpoints and has strong robustness for the prediction of hidden key points. It is very suitable for keypoint detection in complex-occlusion situations.

Inspired by the above idea of latent geometric continuity of key points, we introduce the hypothesis of continuity of translation and rotation transformation behavior of vehicle objects between video frames. For video data, we no longer simply extract the vehicle appearance features of single frame or multiple keyframes but explore the constraint mechanism of latent geometric continuity and appearance features and establish the latent geometric continuity appearance model of vehicle objects. Specifically, the deep convolutional neural network (DCNN) is first used to extract the spatio-temporal information of the object, and then the multi-task direct loss functions of object category, translation, rotation, size, velocity, orientation, and the relative loss functions of object translation and rotation transformation between frames are designed to extract the robust latent geometric continuity apparent model.

### 4.2. Complex Motion Habit Mining and Driving Trajectory Constraint Method

Some researchers have attempted to extract the previous driving trajectories of vehicle objects in video data to assist their apparent features for 3D object motion detection and tracking. However, in complex motion scenes, the motion of the target vehicle is intertwined with the behavior of the interference object, and its motion driving law changes complex during occlusion, resulting in a decrease in tracking accuracy.

Video action understanding includes action recognition, temporal action detection, and video summarization generation, which is a research hotspot in the field of computer vision. Researchers believe that perceiving dynamic behavior may be a major advance in how machines understand the world [22], and focusing on regional spatio-temporal information between video frames helps to recognize events in videos [23]. Therefore, it is possible to enhance the performance of 3D object detection and tracking models by studying the past driving data of each vehicle and discovering deep behavior knowledge in the motion structure characteristics of the vehicle object.

Inspired by the above idea of video dynamic behavior perception, this study uses the previous driving data of each vehicle to find the complex motion habits of the target vehicle and constrain its driving trajectory during the occlusion period so as to improve the accuracy of target tracking. Specifically, based on the latent geometric continuity appearance model, the occupancy ratio of nearby interference objects in the three-dimensional grid near the target vehicle is searched in detail, and the relative translation and relative rotation positive loss function between the target vehicle and its neighboring interference objects is constructed to learn vehicle interaction behavior, determine the complex motion habits of the target vehicle and constrain its driving trajectory during the occlusion period, and improve target tracking accuracy.

### 4.3. Collaborative Calibration Method for Vehicle forward and Reverse Motion Trajectories

Existing 3D object-motion-detection and -tracking methods generally enhance the performance of detection and tracking algorithms through the appearance and motion features of the target object. However, in severe-occlusion scenarios, the target vehicle is occluded to a high degree and for a long time, and the discrimination between its previous appearance features and motion features is significantly reduced. Therefore, the target vehicle may have a variety of complex motion laws in this scenario, which limits the tracking accuracy.

Video coding standards such as H.264 [20] and HEVC [21] classify video frames into intra-frame coding frames, forward prediction coding frames, and bidirectional prediction interpolation coding frames. The bidirectional prediction interpolation coding frame is obtained by predicting its adjacent preceding and following frames, which effectively compresses the temporal redundant information between video frames. The compression rate is usually higher.

Inspired by the above bi-directional inter-frame compression algorithm, this study proposes a collaborative calibration method for vehicle forward and reverse motion trajectories. In the severe-occlusion scene, after the target vehicle gets out of the occlusion, the motion trajectories of each vehicle in the occlusion period are inversely estimated, and the target vehicle’s forward estimated trajectory is matched to realize the collaborative calibration of the target vehicle’s motion trajectory. The tracking results are optimized by combining the artificial fish swarm algorithm. Specifically, compared with the current target vehicle in severe-occlusion scene, its apparent features and motion features are easier to extract after getting out of severe occlusion. Therefore, this study constructs the relative offset and relative attitude reverse loss function of the target vehicle and its adjacent interference object and estimates the motion trajectory of each vehicle in the occlusion period. The forward and reverse trajectory-matching loss function of the vehicle is designed to collaboratively calibrate the target vehicle trajectory, and the tracking result is optimized by combining the artificial fish swarm algorithm.

### 4.4. Apparent Models of Latent Geometric Continuity

**Figure 3:**
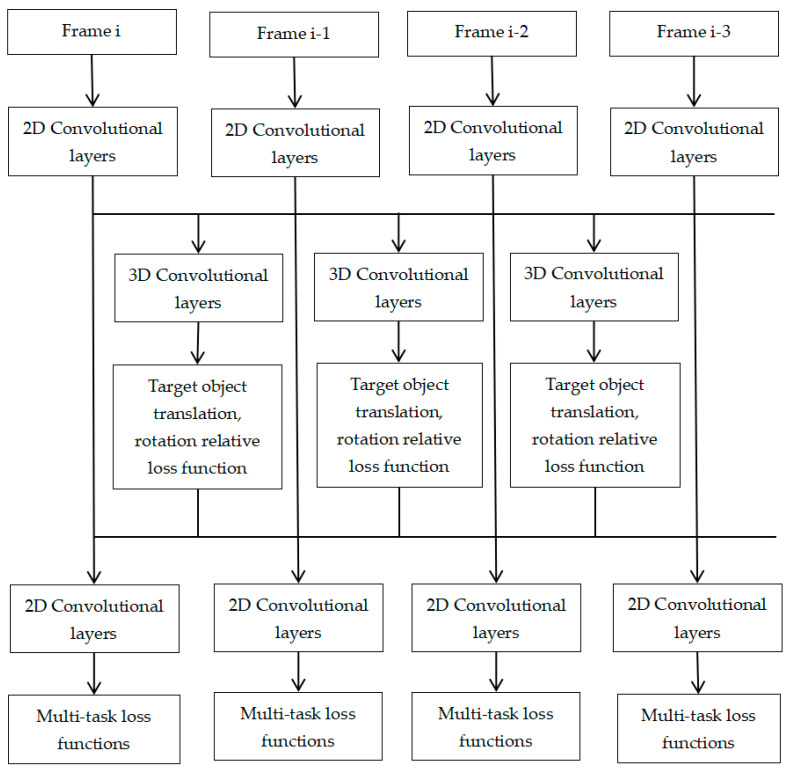
Latent geometric continuity appearance model.

In this study, a 3D convolutional neural network (including a 2D convolutional layer and a 3D convolutional layer) is used to extract the spatio-temporal information of the object in the image sequence. Then, a multi-task direct loss function for object category, translation, rotation, size, speed, and direction, as well as a relative loss function for object translation and rotation transformation between frames, are designed to obtain robust apparent features of potential geometric continuity. The technical route is shown in Figure 3.

The two-dimensional convolutional layer and three-dimensional convolutional layer alternating depth convolutional neural network structure were designed. The key point heat map was generated through the two-dimensional convolutional layer, and each frame corresponded to a set of key point heat maps to predict the center point of each object. Then, the depth information, size, posture, and other attributes of the object were inferred through the surrounding features of the center point. The 3D convolutional layer is used to construct the geometric constraint model of the object space between frames, and the relative transformation loss function of the translation and rotation of the object between frames is designed to obtain the robust apparent feature of potential geometric continuity.

1. Architecture Design of 3D Convolutional Neural Networks;

In the study of 3D object motion detection and tracking in complex-occlusion scenes, an easy way to think of is to treat each frame of video data as a still image and use 2D convolutional neural network to extract the apparent features of each frame of the object. However, this method will cause great variation in the feature distribution of the target object in complex-occlusion scenes caused by neighboring moving objects. It is difficult to obtain apparent features with high discrimination. Therefore, this study introduces the continuity assumption of translation and rotation transformation behavior of vehicle objects between video frames, explores the constraint mechanism between latent geometric continuity and appearance features, and establishes the latent geometric continuity appearance model of vehicle objects. Specifically, a two-dimensional convolutional layer and a three-dimensional convolutional layer alternating deep convolutional neural network structure are designed to generate multiple information channels from adjacent video frames, and convolution and downsampling are performed in each channel, respectively, to extract the apparent features of the target vehicle in the temporal and spatial dimensions.

In the input of 3D convolutional neural network (including 2D convolutional layer and 3D convolutional layer), I ∈ R^W×H×3×N^ represents the image sequence; W and H represent the width and height of the image, respectively; and N represents the number of images. nuScenes [1] dataset consists of 1000 scenes, each scene contains 20 s of video data, and keyframes are sampled at the frequency of 2 Hz for annotation. According to the research results of Russakovsky et al. [22], about 3 s of video data can provide sufficient context information. The computational requirements are kept low; therefore, the input of the 3D convolutional neural network is constructed by 7 keyframes; that is, N = 7.

2. Keypoint-based Multi-task Convolutional Neural Networks;

Compared with the two-stage 3D object detection, the 3D object detection based on keypoints does not require post-processing steps such as non-maximum suppression, which is more efficient and has a good speed and accuracy trade-off. In this study, we propose a keypoint-based multi-task convolutional neural network, which uses the keypoint heatmap to directly regress the object pose; translation; 3D bounding box center point; length; width; and height; as well as motion speed, confidence, and other attributes. The framework outputs a set of keypoint heatmaps. These heatmaps are low-resolution heatmaps, Ŷ ∈ ${\left[0,1\right]}^{\frac{W}{M}\times \frac{H}{M}\times C}$, with an output stride M = 4, where M represents the output step; W and H represent the width and height of the input image, respectively, according to [8,24,25,26]; M = 4 is set as the factor of sub-sampling output heatmap; and C represents the object category of the current frame. nuScenes [1] dataset C = 23. The heatmap ${Ŷ}_{x,y,c}$ = 1 corresponds to a detected keypoint, and ${Ŷ}_{x,y,c}$ = 0 corresponds to the background.

According to the existing keypoint prediction network [8,27], for each truth keypoint p ∈ R^2^ of c class objects, its low-resolution equivalent representation $\tilde{p}$ = $\left\lfloor \frac{p}{M}\right\rfloor$ is calculated, and then a Gaussian kernel is used to combine all sample keypoints into a keypoint heat map ${\hat{Y}}_{xyc}\;$= $exp\left(-\left({\left(x-{\tilde{p}}_{x}\right)}^{2}+{\left(y-{\tilde{p}}_{y}\right)}^{2}\right)/2\sigma_{p}^{2}\right)$, where σ_p_ denotes the object size adaptive standard deviation. The network training objective is a pixel-wise logistic regression object center loss function with the following form, as shown in Equation (1):

(1) $$L_{k}=\frac{-1}{N}\sum_{xyc}\left\{\begin{array}{ll} {\left(1-{\hat{Y}}_{xyc}\right)}^{\alpha }log{\hat{Y}}_{xyc} & if\;{\hat{Y}}_{xyc}=1\\ {\left(1-{\hat{Y}}_{xyc}\right)}^{\beta }{\left({\hat{Y}}_{xyc}\right)}^{\alpha }log\left(1-{\hat{Y}}_{xyc}\right) & otherwise \end{array}\right.,$$

where α = 2 and β = 4 are the hyperparameters of the focal loss function, and N is the number of keypoints in image *I*. Also, to recover the discretization error, we add a center point local translation Ô ∈ $R^{\frac{W}{M}\times \frac{H}{M}\times 2}$, trained by the L1 loss function, as shown in Equation (2):

(2) $$L_{off}=\frac{1}{N}\sum_{p}\left|{Ô}_{\tilde{p}}-\left(\frac{p}{M}-\tilde{p}\right)\right|,$$

After predicting the object center translation information, we add a branch to the network to predict the object center depth information d by introducing the output transformation d = 1/σ($\hat{d}$) − 1 (σ is the sigmoid function) proposed by Eigen et al. [28]. The L1 loss is used to compute the depth information D from the additional output channels $\hat{D}$ ∈ ${\left[0,1\right]}^{\frac{W}{M}\times \frac{H}{M}}$. Similarly, the pose quaternion R; translation matrix t; width; height; and length S = S_w_, S_h_, S_l_; object orientation O; and object velocity V attributes of the 3D object bounding box are predicted from the corresponding additional network branch head.

3. Interframe target object space geometric constraint model.

In the complex-occlusion scene caused by neighboring moving objects, the apparent features of the target object change significantly between frames. In order to overcome the problem wherein the large variation of the feature distribution law leads to the large search space of feature matching between frames, we start from the scene of simple motion of the target object. By framing between target object via translation, rotation transformation relative loss function *Δ*[R^i,i−1^| t^i,i−1^], *Δ*[R^i−1,i−2^| t^i−1,i−2^], *Δ*[R^i−2,i−3^| t^i−2,i−3^] object space geometric constraint model builds frames to explore object translation and rotation transformation potential geometry continuity between frames. Thus, a robust object appearance model is obtained. There is a relative shift between the target frame, rotating *Δ*[R^i,i−1^| t^i,i−1^] by the ith frame [R^i^|t^i^] with the i − 1 frame [R^i−1^|t^i−1^] reasoning, as shown in Formula (3):

(3) $$\Delta \left[R^{i,i-1}|t^{i,i-1}\right]=\left[R^{i-1}|t^{i-1}\right]{\left[R^{i}|t^{i}\right]}^{-1},$$

Here, [R^i^|t^i^]^−1^ is the inverse [R^i^|t^i^], and *Δ*[R^i,i−1^|t^i,i−1^] has the following form, as shown in Formula (4):

(4) $$\left[\begin{array}{cc} R^{i,i-1} & t^{i,i-1}\\ 0 & 1 \end{array}\right],$$

where R^i,i−1^ is the 3D rotation matrix, and t^i,i−1^ is the translation matrix. By the same token, the *Δ*[R^i−1,i−2^|t^i−1,i−2^], as *Δ*[R^i−2,i−3^|t^i−2,i−3^] has a similar form, the said translation, with relative rotation change between the object frame.

Finally, we design the latent geometric continuity loss function L^i,i−1^ to measure the relative error of the object 3D bounding box corners between frame i and frame i − 1, as shown in Equation (5):

(5) Lgeoi,i−1=1M∑jM∥(R^i,i−1xj+t^i,i−1)−(Ri,i−1xj+ti,i−1)∥,

where x_j_ represents the *j*th corner of the M 3D bounding box corners, and $\hat{R}$^i,i−1^ and $\hat{t}$^i,i−1^ represent the relative rotation and translation prediction results between frames i and i − 1, respectively. Then, R^i,i−1^ and t^i,i−1^ correspond to the true value relative rotation matrix and relative translation matrix of the object between frames i and i − 1. Similarly, other neighboring inter-frame latent geometric continuity loss functions have similar forms.

### 4.5. Complex Motion Habit-Mining and Driving-Trajectory Constraint Method

**Figure 4:**
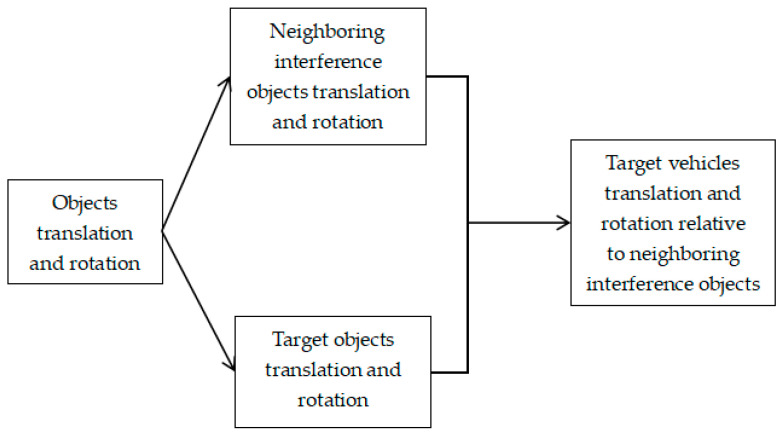
Using the previous driving data of each vehicle, the complex motion habits of the target vehicle are mined, and its driving trajectory during the occlusion period is constrained.

In complex motion scenes, the behavior of the target vehicle is intertwined with the behavior of nearby objects, which leads to complex changes in the movement and driving law of the target vehicle, and the tracking accuracy decreases sharply. This study constructs the positive loss function of relative offset and relative attitude between the target vehicle and its adjacent interference objects by searching the occupancy ratio of neighboring interference objects in the three-dimensional grid near the target vehicle, learns the knowledge of vehicle interaction behavior, finds the complex motion habits of the target vehicle, and restricts its driving trajectory during the occlusion period accordingly so as to improve the target tracking accuracy. The method is shown in Figure 4.

The 3D-object-motion detection result $E_{c,\;j}^{i}$ = {$R_{c,\;j}^{i}$, $t_{c,\;j}^{i}$, C*_xyz,c,j_*, S*_whl,c,j_*, $O_{c,\;j}^{i}$, $V_{c,\;j}^{i}$} represents the rotation, translation, center, size, direction, and speed of the JTH object of class c. It is possible to use $E_{c,\;j}^{i}$ to search the neighbor interference objects of the target object, excavate the complex motion habits of the vehicle object, and constrain the driving trajectory. The specific steps are as follows:

The first step: fine-grained search of target object neighbor interference objects. Firstly, a 3D grid Bγ is set around the target object, and its length, width, and height are γ. Then, the intersection IoU of the 3D bounding box of other objects in the image and the 3D grid Bγ is calculated. If the IoU exceeds 30%, the object is judged to be the nearest interference object of the target object.

Step 2: Introduce [29,30] to calculate the relative translation and rotation residuals between the target object and its neighbors, as shown in Equation (6):

(6) $$\Delta (R_{tar}^{i},R_{j}^{i})=\frac{{∥log(R_{tar}^{i^{T}}R_{j}^{i})∥}_{F}}{\sqrt{2}},$$

where $R_{tar}^{i}$ and $R_{j}^{i}$ denote the pose of the target object and the *j*th object in its immediate neighborhood, respectively. Translation change residuals are correlated.

*Δ*($t_{tar}^{i}$, $t_{j}^{i}$) is then calculated by smoothing L1 loss [31].

Step 3: Design the loss function $L_{rela}^{i}$ to calibrate the 3D-object-detection and -tracking results, which has the following form, as shown in Equation (7):

(7) $$L_{rela}^{i}=\lambda^{i}\frac{1}{N}\sum_{j}^{N}\left(\Delta \left(R_{tar}^{i},R_{j}^{i}\right)+\Delta \left(t_{tar}^{i},t_{j}^{i}\right)\right),$$

As time goes by, the influence of the interactive motion characteristics of vehicles on the current frame i gradually increases. Therefore, in this part of the technical route, the regularization parameter needs to be constrained to increase to calibrate the 3D object’s motion detection and tracking.

### 4.6. Collaborative Calibration and AFSA Method for Vehicle forward and Reverse Motion Trajectories

In severe-occlusion scenarios, the target vehicle is usually severely occluded for a long time, and the discrimination between its previous appearance features and motion features is reduced. As a result, there may be a variety of personalized motion laws for the target vehicle during the continuous-occlusion period, which greatly restricts the tracking accuracy.

**Figure 5:**
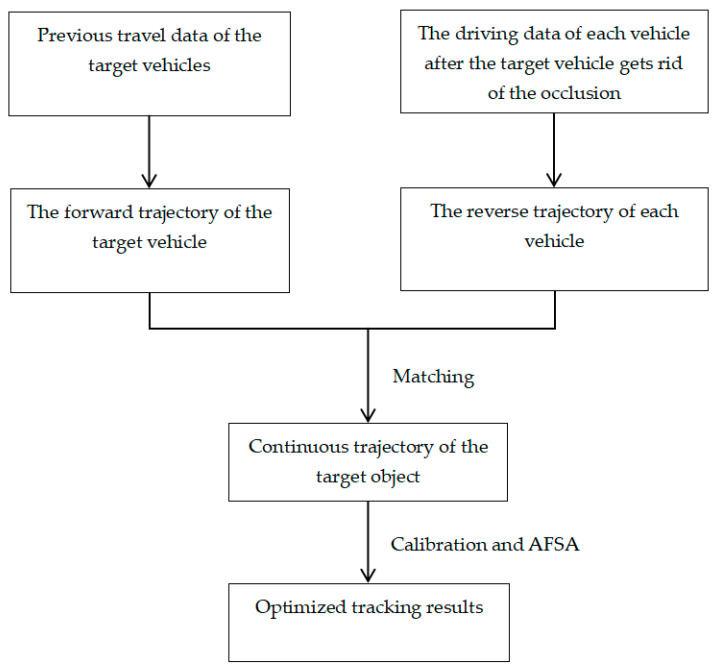
The vehicle's forward and reverse trajectory matching loss functions were designed to collaboratively calibrate the target vehicle trajectory.

Compared with the target vehicle under continuous occlusion, the apparent features and motion features of the target vehicle are easier to extract after the target vehicle gets rid of the occlusion. Therefore, it is possible to construct the reverse loss function of the relative offset and attitude between the target vehicle and its adjacent interference objects, to reverse the motion trajectory of each vehicle in the occlusion period, and to design the matching loss function of the forward and reverse motion trajectory of the vehicle. The target vehicle trajectory is coordinately calibrated, and the tracking results are optimized by combining the artificial fish swarm algorithm. The specific method is shown in Figure 5.

Using the Siamese convolutional network structure [32] and its variants [33,34,35], this study constructs a convolutional neural network for matching the forward and reverse trajectory of the vehicle and designs a joint calibration loss function, as shown in Equation (8):

(8) $$\begin{array}{c} L_{cb}\left(V_{p},V_{n}\right)=-1/\left(MN\right)\sum_{i}^{M}\sum_{j}^{N}logprob\left(vp_{i},vn_{i}\right)\\ prob\left(vp_{i},vn_{j}\right)=e^{vp_{i}}/\left(e^{vp_{i}}+e^{vn_{j}}\right) \end{array},$$

Here, M represents the number of driving trajectory data before the occlusion of the target object, and N represents the number of driving trajectories after the occlusion of the target object. L_cb_(V_p_, V_n_) is used to calibrate the continuity of the vehicle motion trajectory, output the forward motion cue and reverse motion cue of the target vehicle, assist the potential geometric continuity apparent feature, and realize accurate 3D object detection and tracking. 

Furthermore, we use an adaptive step size artificial fish swarm algorithm to achieve high convergence accuracy while maintaining good convergence speed. Its adaptive step size is shown in Equation (9).

(9) $$p_{i}=\left\{\begin{array}{cc} p-\alpha \cdot logD_{i}-\beta \cdot i\cdot R, & D_{i}\neq 0;\\ \delta \cdot p, & \sum_{i}^{i-3}D_{i}=0,i>4;\\ K, & D_{i}=0. \end{array}\right.,$$

In the formula, p_i_ represents the step size of the artificial fish iteration at generation i; p is the initial value of the step size. α is the decrease rate factor; D_i_ represents the decline rate of artificial fish in the ith generation; β represents the random number factor; R represents a normally distributed random number that produces a [–1, 1]. Let δ·p, K denote the dynamic adjustment strategy adopted by the step size when the drop rate is zero or the drop rate is zero continuously, respectively.

Inspired by the artificial fish swarm algorithm, we design a batch triple loss function $L_{tri}$ to mine the spatio-temporal dependency knowledge in the cooperative changes of object position, direction, and speed, which provides key support for the tracked target object $n_{i}^{t}$ in t frames to find the object $n_{j}^{t+1}$ to be matched in t + 1 frames with similar changes of its motion position, direction, and speed. The batch triple loss function $L_{tri}$ is shown in Equation (10):

(10) $$L_{tri}=max(||n_{i,l}^{t^{\prime }}-n_{j,l}^{t+1^{\prime }}||-min_{d_{s}\in D}(||n_{i,l}^{t^{\prime }}-n_{s,l}^{t+1^{\prime }}||)-min_{b_{r}\in B}(||n_{r,l}^{t^{\prime }}-n_{j,l}^{t+1^{\prime }}||)+\alpha ,0),$$

where α is the boundary of the batch triple loss function, and $n_{s,l}^{t+1\prime }$ is the interactive collaborative feature representation of the position, direction, and velocity of the object s to be matched in t + 1 frames. In the formula, $n_{s,l}^{t+1\prime }$ denotes the node that is different from $n_{j,l}^{t+1\prime }$ and $n_{i,l}^{t\prime }$ in frame t + 1, and similarly, $n_{r,l}^{t\prime }$ denotes the node that is different from $n_{j,l}^{t+1\prime }$ and $n_{i,l}^{t\prime }$ in frame t. By constructing the above batch triple loss function, the supervised graph neural network mines the spatio-temporal dependency knowledge of the position, direction, and speed of the moving object, which provides an important support for the construction of graph neural network based on multi-source feature interweaving.

The cooperative calibration and artificial fish swarm algorithm jointly complete the optimization of vehicle motion trajectory and realize the exploration of a new way to support L5 autonomous driving on real roads with intelligent perception technology.

## 5. Experiment

### 5.1. Experimental Data

nuScenes [1] dataset is used for network training, validation, and testing in this chapter. The nuScenes [1] dataset consists of 1000 scenes, of which 700 scenes are used for training, 150 scenes are used for validation, and 150 scenes are used for testing. Each scene comprises 20 s, and six cameras sampled at 2 Hz can form slightly overlapping 360° panoramic viewpoints. Each scene contains 40 keyframes; thus, there is a total of 168 000 training images, 36 000 validation images, and 36 000 test images.

### 5.2. Experimental Environment

In this experiment, the performance of different algorithms is verified on Lenovo thinkstation workstation, and the platform configuration used in the experiment is shown in Table 1.

### 5.3. Experimental Result

In this experiment, the method in this chapter will be compared with the CenterTrack method on the validation set and test set of nuScenes [1] dataset. The nuScenes [1] validation set provides annotations in seven categories for 3D object detection and tracking tasks and consists of 150 scenes, each of which contains 20 s of autonomous driving video footage, each of which contains 40 keyframes. In this study, on the nuScenes [1] validation set, the comparative results of the deep convolutional neural network based on driving behavior knowledge discovery and CenterTrack in the evaluation indicators such as AMOTA (Average Multi-Object Tracking Accuracy) and AMOTP (Average Multi-Object Tracking Precision) are verified, as shown in Table 2.

nuScenes [1] test set is different from the validation set. The test set does not provide public annotations and only provides the comparison results of 3D target detection and tracking evaluation indicators such as AMOTA, AMOTP, MT, and ML through the online valAi cloud computing platform. The experimental comparison results are shown in Table 3. They show that the effect of our method is improved compared with the existing methods when ASFA is not added, and there is a slight improvement when the ASFA algorithm is added to our method.

**Figure 6:**
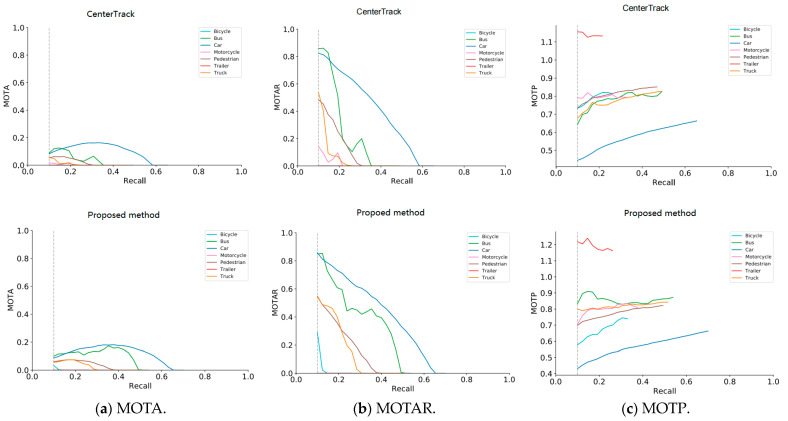
Plot of MOT A, MOTAR, and MOTP−recall curve for 3D object tracking on nuScenes validation set.

Based on the experimental results, Figure 6 shows the comparison results of the proposed method and CenterTrack method on the key indicators MOTA, MOTAR(Multi-Object Tracking Accuracy and Robustness), and MOTP of the 3D object detection and tracking challenge on the nuScenes [1] validation set. The MOTA−recall and MOTAR−recall graphs show that the lower dark blue curve, green curve, and orange curve are significantly better than the upper curve of the same category (the higher the MOTA and MOTAR, the better the effect). Combined with the AMOTA and AMOTAR data in Table 2, it is verified that the motion feature representation method based on behavior analysis proposed in this chapter can effectively constrain the motion trajectory of the target in the occlusion period and has a more stable tracking effect compared with the CenterTrack method. As can be seen in the MOTP − Recall graph, the lower blue curve and light blue curve are significantly better than the upper curve of the same category (the lower the MOTP, the better the effect), combined with the average AMOTP = 1.485 of the lower curve and the average AMOTP = 1.543 of the upper curve. It is verified that the method proposed in this chapter can measure the size, position, and orientation of the 3D bounding box of the target more accurately than the CenterTrack method.

**Figure 7:**
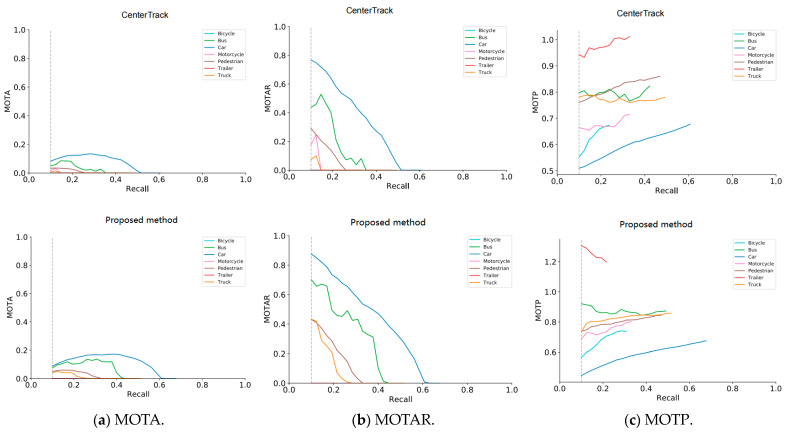
Plot of MOT A, MOTAR, and MOTP−recall curve for 3D object tracking on nuScenes test set.

Figure 7 shows the comparison results between the method proposed in this chapter and the CenterTrack method on the key indicators MOTA, MOTAR, and MOTP of the 3D target tracking challenge in the nuScenes [1] test set. AMOTA is the average MOTA of the seven categories of bicycle, bus, car, motorcycle, pedestrian, trailer, and truck in the test set, which is the primary reference for the performance ranking of nuScenes algorithm. Combined with the AMOTA and AMOTAR data in Table 3, it is further verified on the test set that the motion feature representation method based on behavior analysis proposed in this chapter has a more stable tracking effect than the CenterTrack method. In complex road traffic scenes where the target object is occluded or truncated, and in bad weather conditions such as rain and snow, it has higher robustness and is more accurate in the determination of the size, position, and orientation of the target 3D bounding box.

## 6. Conclusions and Future Work

Starting with the complex motion of the target object, this study examines the interleaved motion behavior of the target and its neighboring interference objects. Using the previous motion data of the target, the knowledge of the target motion behavior is mined to constrain the motion trajectory of the target in the occlusion period. Compared with the existing methods, the deep convolutional neural network is supervised by the 2D offset distance of the target center. The motion feature representation method based on behavior analysis helps the network to stably track the 3D bounding box of the target in the occlusion period and, after getting out of the occlusion, accurately determine its size, position, and orientation. The artificial fish swarm algorithm with an adaptive step size provides optimized motion path planning. Together, they can provide reliable motion planning clues for automatic driving systems and assistant driving systems to ensure driving safety.

Although this study provides a good improvement in AMOT A, MOT P, and other evaluation indicators compared with the current optimal 3D object-detection and -tracking algorithm, there is still room for further improvement and improvement in future work. In complex road traffic scenes, from the perspective of the lighting environment, on the one hand, the lighting conditions of the target vehicle often change due to objective factors such as entering and leaving tunnels, tree shade, and so on. The significant variation of local lighting conditions of the target object presents significant challenges in the feature extraction of the target object and the correlation between the target object and its frames. Therefore, future work will focus on the condition of the large variation of target object characteristics caused by the sudden change of local illumination conditions during the daytime driving of the target vehicle. It is important to study 3D target detection and tracking under the scene of sudden changes in the local illumination intensity during daytime.

## Figures and Tables

**Table 1:** Experimental platform parameters.

	Computer Configuration
CPU	Intel Core i7-7700
GPU	NVIDIA GeForce RTX 3070
Memory	32 G
Operating System	Ubuntu 16.04

**Table 2:** Comparison results using the validation set.

	AMOTA	AMOTP	MOTAR	MT	ML	IDS	FP	FN
CenterTrack	0.068	1.543	0.332	538	4231	2962	15,733	73,573
Ours	0.098	1.49	0.297	635	4080	2415	16,738	70,544

**Table 3:** Comparison results of the test set.

	Mapillary + AB3D	GDLG	CenterTrack	CaTracker	Buffalo Vision	ProTracker	Ours (without AFSA)	Ours (with AFSA)
AMOTA	0.018	0.045	0.046	0.053	0.059	0.072	0.072	0.073
AMOTP	1.790	1.819	1.543	1.611	1.490	1.628	1.490	1.489
MOTAR	0.091	0.242	0.231	0.168	0.244	0.256	0.355	0.349
MOTA	0.020	0.050	0.043	0.041	0.048	0.058	0.069	0.069
MOTP	0.903	0.970	0.753	0.779	0.781	0.785	0.753	0.727
MT	499	448	573	769	675	729	855	867
ML	4700	4094	5235	4587	4835	4808	4710	4713
FAF	356.753	211.536	75.945	84.284	81.302	60.914	58.919	58.471
TP	25,943	29,198	26,544	33,937	32,325	32,245	33,957	33,971
FP	113,596	40,742	17,574	24,574	26,003	17,957	19,984	19,974
FN	83,202	78,327	89,214	81,171	83,070	83,314	81,978	81,974
IDS	10,420	12,040	3807	4457	4170	4006	3902	3841
FRAG	4403	4138	2645	3169	3157	2935	2762	2744
TID	1.414	4.705	2.057	1.953	2.177	2.306	2.301	2.127
LGD	3.351	5.926	3.819	3.765	4.088	4.023	4.053	4.065

## Data Availability

The original contributions presented in the study are included in the article, further inquiries can be directed to the corresponding authors.

## References

[1] Singh S. (2023). Traffic Safety Facts Critical Reasons for Crashes Investigated in the National Motor Vehicle Crash Causation Survey.

[2] Geiger A., Lenz P., Urtasun R. Are we ready for autonomous driving? the kitti vision benchmark suite. Proceedings of the 2012 IEEE Conference on Computer Vision and Pattern Recognition.

[3] Huang X., Cheng X., Geng Q., Cao B., Zhou D., Wang P., Lin Y., Yang R. The apolloscape dataset for autonomous driving. Proceedings of the IEEE Conference on Computer Vision and Pattern Recognition Workshops.

[4] He J., Chen Y., Wang N., Zhang Z. 3D Video Object Detection with Learnable Object-Centric Global Optimization. Proceedings of the 2023 IEEE/CVF Conference on Computer Vision and Pattern Recognition.

[5] Xiang Y., Schmidt T., Narayanan V., Fox D. Posecnn: A convolutional neural network for 6d object pose estimation in cluttered scenes. Proceedings of the 2021 IEEE International Conference on Image Processing.

[6] Li B., Ouyang W., Sheng L., Zeng X., Wang X. Gs3d: An efficient 3d object detection framework for autonomous driving. Proceedings of the IEEE Conference on Computer Vision and Pattern Recognition.

[7] Arnold E., Al-Jarrah O.Y., Dianati M., Fallah S., Oxtoby D., Mouzakitis A. (2019). A survey on 3d object detection methods for autonomous driving applications. IEEE Trans. Intell. Transp. Syst..

[8] Zhou X., Wang D., Krähenbühl P. (2019). Objects as points. arXiv.

[9] Zhou X., Koltun V., Krähenbühl P. (2020). Tracking Objects as Points. arXiv.

[10] Wu Y. Monocular Instance Level 3D Object Reconstruction based on Mesh R-CNN. Proceedings of the 2020 5th International Conference on Information Science, Computer Technology and Transportation.

[11] Zheng X., Chen F., Lou L., Cheng P., Huang Y. (2022). Real-Time Detection of Full-Scale Forest Fire Smoke Based on Deep Convolution Neural Network. Remote Sens..

[12] Girshick R., Donahue J., Darrell T., Malik J. Rich feature hierarchies for accurate object detection and semantic segmentation. Proceedings of the IEEE Conference on Computer Vision and Pattern Recognition.

[13] Chabot F., Chaouch M., Rabarisoa J., Teuliere C., Chateau T. Deep manta: A coarse-to-fine many-task network for joint 2d and 3d vehicle analysis from monocular image. Proceedings of the IEEE Conference on Computer Vision and Pattern Recognition.

[14] Mousavian A., Anguelov D., Flynn J., Kosecka J. 3d bounding box estimation using deep learning and geometry. Proceedings of the IEEE Conference on Computer Vision and Pattern Recognition.

[15] Chen Y., Liu S., Shen X., Jia J. (2020). DSGN: Deep Stereo Geometry Network for 3D Object Detection. arXiv.

[16] Zhao Q., Zhang L., Liu L., Shuchang B., Yong C., Han L. Swarm Motion of Underwater Robots Based on Local Visual Perception. Proceedings of the 2023 8th International Conference on Automation, Control and Robotics Engineering.

[17] Li X., Xia X., Hu Z., Han B., Zhao Y. Intelligent Detection of Underwater Fish Speed Characteristics Based on Deep Learning. Proceedings of the 2021 5th Asian Conference on Artificial Intelligence Technology.

[18] Suwajanakorn S., Snavely N., Tompson J.J., Norouzi M. Discovery of latent 3d keypoints via endto-end geometric reasoning. Proceedings of the Advances in Neural Information Processing Systems.

[19] Ji S., Xu W., Yang M., Yu K. (2012). 3D convolutional neural networks for human action recognition. IEEE Trans. Pattern Anal. Mach. Intell..

[20] Ji S., Xu W., Yang M., Yu K. (2003). Overview of the H. 264/AVC video coding standard. IEEE Trans. Circuits Syst. Video Technol..

[21] Sullivan G.J., Ohm J.R., Han W.J., Wiegand T. (2012). Overview of the high efficiency video coding (HEVC) standard. IEEE Trans. Circuits Syst. Video Technol..

[22] Knight W. The Next Big Step for AI? Understanding Video. https://www.technologyreview.com/s/609651/the-next-big-step-for-ai-understanding-video/.

[23] Mozaffari S., Al-Jarrah O.Y., Dianati M., Jennings P., Mouzakitis A. (2019). Deep Learning-based Vehicle Behaviour Prediction for Autonomous Driving Applications: A Review. arXiv.

[24] Cao Z., Simon T., Wei S.E., Sheikh Y. Realtime multi-person 2d pose estimation using part affinity fields. Proceedings of the IEEE Conference on Computer Vision and Pattern Recognition.

[25] Newell A., Yang K., Deng J. Stacked hourglass networks for human pose estimation. Proceedings of the European Conference on Computer Vision.

[26] Papandreou G., Zhu T., Kanazawa N., Toshev A., Tompson J., Bregler C., Murphy K. Towards accurate multi-person pose estimation in the wild. Proceedings of the IEEE Conference on Computer Vision and Pattern Recognition.

[27] Law H., Deng J. Cornernet: Detecting objects as paired keypoints. Proceedings of the European Conference on Computer Vision (ECCV).

[28] Eigen D., Puhrsch C., Fergus R. Depth map prediction from a single image using a multi-scale deep network. Proceedings of the Advances in Neural Information Processing Systems.

[29] Su H., Qi C.R., Li Y., Guibas L.J. Render for cnn: Viewpoint estimation in images using cnns trained with rendered 3d model views. Proceedings of the IEEE International Conference on Computer Vision.

[30] Tulsiani S., Malik J. Viewpoints and keypoints. Proceedings of the IEEE Conference on Computer Vision and Pattern Recognition.

[31] Girshick R. Fast r-cnn. Proceedings of the IEEE International Conference on Computer Vision.

[32] Kim M., Alletto S., Rigazio L. (2016). Similarity mapping with enhanced siamese network for multiobject tracking. arXiv.

[33] Wang B., Wang L., Shuai B., Zuo Z., Liu T., Luk Chan K., Wang G. Joint learning of convolutional neural networks and temporally constrained metrics for tracklet association. Proceedings of the IEEE Conference on Computer Vision and Pattern Recognition Workshops.

[34] Zhang S., Gong Y., Huang J.B., Lim J., Wang J., Ahuja N., Yang M.H. Tracking persons-of-interest via adaptive discriminative features. Proceedings of the European Conference on Computer Vision.

[35] Moghaddam M., Charmi M., Hassanpoor H. (2022). A robust attribute-aware and real-time multi-target multi-camera tracking system using multi-scale enriched features and hierarchical clustering. J. Real-Time Image Process..

